# Functional coordination of BET family proteins underlies altered transcription associated with memory impairment in fragile X syndrome

**DOI:** 10.1126/sciadv.abf7346

**Published:** 2021-05-19

**Authors:** Seung-Kyoon Kim, Xihui Liu, Jongmin Park, Dahun Um, Gokhul Kilaru, Cheng-Ming Chiang, Mingon Kang, Kimberly M. Huber, Keunsoo Kang, Tae-Kyung Kim

**Affiliations:** 1 Department of Life Sciences, Pohang University of Science and Technology, Pohang, Gyeongbuk 37673, Korea.; 2 Department of Neuroscience, University of Texas Southwestern Medical Center, Dallas, TX 75390, USA.; 3 Simmons Comprehensive Cancer Center, Department of Biochemistry, and Department of Pharmacology, University of Texas Southwestern Medical Center, Dallas, TX 75390, USA.; 4 Department of Computer Science, University of Nevada, Las Vegas, NV 89154, USA.; 5 Department of Microbiology, Dankook University, Cheonan 31116, Korea.

## Abstract

Bromodomain and extraterminal proteins (BET) are epigenetic readers that play critical roles in gene regulation. Pharmacologic inhibition of the bromodomain present in all BET family members is a promising therapeutic strategy for various diseases, but its impact on individual family members has not been well understood. Using a transcriptional induction paradigm in neurons, we have systematically demonstrated that three major BET family proteins (BRD2/3/4) participated in transcription with different recruitment kinetics, interdependency, and sensitivity to a bromodomain inhibitor, JQ1. In a mouse model of fragile X syndrome (FXS), BRD2/3 and BRD4 showed oppositely altered expression and chromatin binding, correlating with transcriptional dysregulation. Acute inhibition of CBP/p300 histone acetyltransferase (HAT) activity restored the altered binding patterns of BRD2 and BRD4 and rescued memory impairment in FXS. Our study emphasizes the importance of understanding the BET coordination controlled by a balanced action between HATs with different substrate specificity.

## Teaser

The coordination of BET family proteins is a feature of the gene regulatory network underlying memory behavior and FXS.

## INTRODUCTION

Bromodomain and extraterminal (BET) proteins are epigenetic readers that share two conserved bromodomains and one extraterminal (ET) domain (*1*, *2*). Among the BET family members, BRD2, BRD3, and BRD4 are ubiquitously expressed, but bromodomain testis-specific (BRDT) expression is limited to testis and oocytes (*2*). As epigenetic readers, BET proteins play a key role in gene transcription through their interactions with chromatin and various transcription regulators (*3*–*5*). Being the most extensively studied family member, BRD4 mediates transcriptional responses by recruiting several transcriptional regulators, including Mediator complex and positive transcription elongation factor b (P-TEFb) to the chromatin regions near the *cis*-regulatory regions such as promoters and enhancers (*1*, *6*–*8*). The chromatin occupancy patterns of BRD4 is largely correlated with histone acetylation, suggesting the importance of the bromodomain-dependent recruitment mechanism, but evidence also supports that BRD4 recruitment could be mediated by direct interactions with various transcriptional regulators (e.g., P-TEFb, Myc/Max, and C/EBPα and C/EBPβ) in either a bromodomain-dependent or bromodomain-independent manner (*9*–*11*).

Pharmacologic inhibition of BET protein function has been a promising therapeutic strategy for several diseases including cancers (*4*, *12*–*16*), inflammation (*17*), and heart failure (*18*). To date, all small-molecule inhibitors of BET proteins including JQ1 commonly target the bromodomain binding pocket (*13*, *15*, *17*, *19*), but the majority of studies have suggested that dysregulation of BRD4 function is the primary effect of pharmacologic BET inhibition (*5*, *7*, *11*, *14*–*16*, *18*–*20*). JQ1 exhibits similar affinities to all major BET proteins (*12*, *13*, *15*, *17*, *19*), which suggests that in principle, JQ1-mediated inhibition could affect the bromodomain-dependent function of any BET family protein that shares the conserved bromodomains. The ubiquitously expressed BET proteins may work redundantly and/or coordinately as they exhibit overlapping genomic-binding profiles at active genes (*12*, *21*) and associate with many common transcriptional regulators (*8*, *13*, *22*). On the other hand, several lines of evidence also point to their nonredundant or selective roles in various biological pathways (*3*, *23*–*28*). Distinctive functionality of BET family proteins might be mediated by different binding preference to the acetylated residues (*15*, *29*, *30*) and additional domains that affect their chromatin binding (*10*, *31*, *32*), differences in interaction partners (*8*, *13*, *22*), and the regulatory capacity for higher-order chromatin architecture (*24*). Nonetheless, the functional distinctions among different BET family members at the molecular level are still poorly understood. This subject would be important for the accurate evaluation of the therapeutic benefits of currently available BET inhibitors as well as the development of more selective drugs that might benefit clinical interventions in a range of diseases.

Recent studies demonstrated that BET proteins are also important for brain function and behavior. JQ1 blocks BRD4 function in neurons, resulting in impairment of transcriptional responses and various forms of learning and memory as well as long-term potentiation (LTP) (*33*, *34*). However, the therapeutic effects of BET inhibition have been somewhat incongruous. While treatment of wild-type (WT) mice with a brain-penetrable inhibitor, I-BET858, impairs neuronal gene expression programs and promotes the development of autism-like behaviors (*35*), JQ1 improves memory performance of WT mice in another study (*36*). In a fragile X mental retardation 1 (*Fmr1*) knockout (KO) mouse, a model of autism spectrum disorder, fragile X syndrome (FXS), the level of BRD4 protein is elevated in the brain with widespread changes in chromatin regulation and aberrant gene expression (*37*). JQ1 treatment alleviates FXS-related symptoms. JQ1 also ameliorates Alzheimer’s disease phenotypes in two different mouse models, but its effect in learning and memory deficits are varied (*36*, *38*). In the case of a mouse model for Huntington’s disease (HD), JQ1 exacerbates the dysregulation of HD-related symptoms while enhancing motor performance in WT mice (*39*). Although these studies illustrate the functional relevance of BET inhibition to brain function and related diseases, the molecular changes of individual BET family members caused by small-molecule BET inhibition in the brain have not been investigated.

Sensory experience–evoked neuronal activity critically underlies brain development and function by inducing transcription from a specific set of genes called activity-induced genes. Activity-dependent gene expression is necessary for the long-lasting form of synaptic plasticity, which is generally considered as the cellular correlate for cognitive behaviors (*40*). Transcriptional response to neuronal activity occurs in waves such that a distinctive set of genes is induced at different time windows following evoked activity. Immediately early genes such as *Arc* and *Fos* belong to the first responder group that is rapidly induced by neuronal activity, and their induction is critical for subsequent gene expression and behavior such as learning and memory. Disruption of activity-dependent gene expression programs has been linked to various brain disorders including autism. Here, we dissect the functions of all three major BET family proteins, BRD2/3/4 in activity-dependent gene expression and long-term memory (LTM). We found that all three BET family proteins participated in activity-dependent gene expression. Despite significant overlaps between their binding sites, individual BET family members exhibited notable differences in the sensitivity to inhibition of the BET bromodomain and CBP/p300 histone acetyltransferase (HAT) activity, recruitment kinetics, and protein-protein interactions. All three BET proteins are also involved in LTM and pathological aspects of FXS. Notably, our study uncovered that a small-molecule inhibitor of CBP/p300 HAT activity, C646, altered the chromatin binding patterns of BRD2/3/4 in a manner that can correct the abnormal BET occupancy patterns caused by *Fmr1* loss of function. Together, our data indicate that BET family proteins do not simply act in redundancy, but instead, they coordinate with each other in a hierarchical manner during activity-dependent gene induction, and disruption of the coordinated BET network could contribute to the development of FXS.

## RESULTS

### BET inhibition affects neuronal activity–induced gene expression

**Fig. 1:**
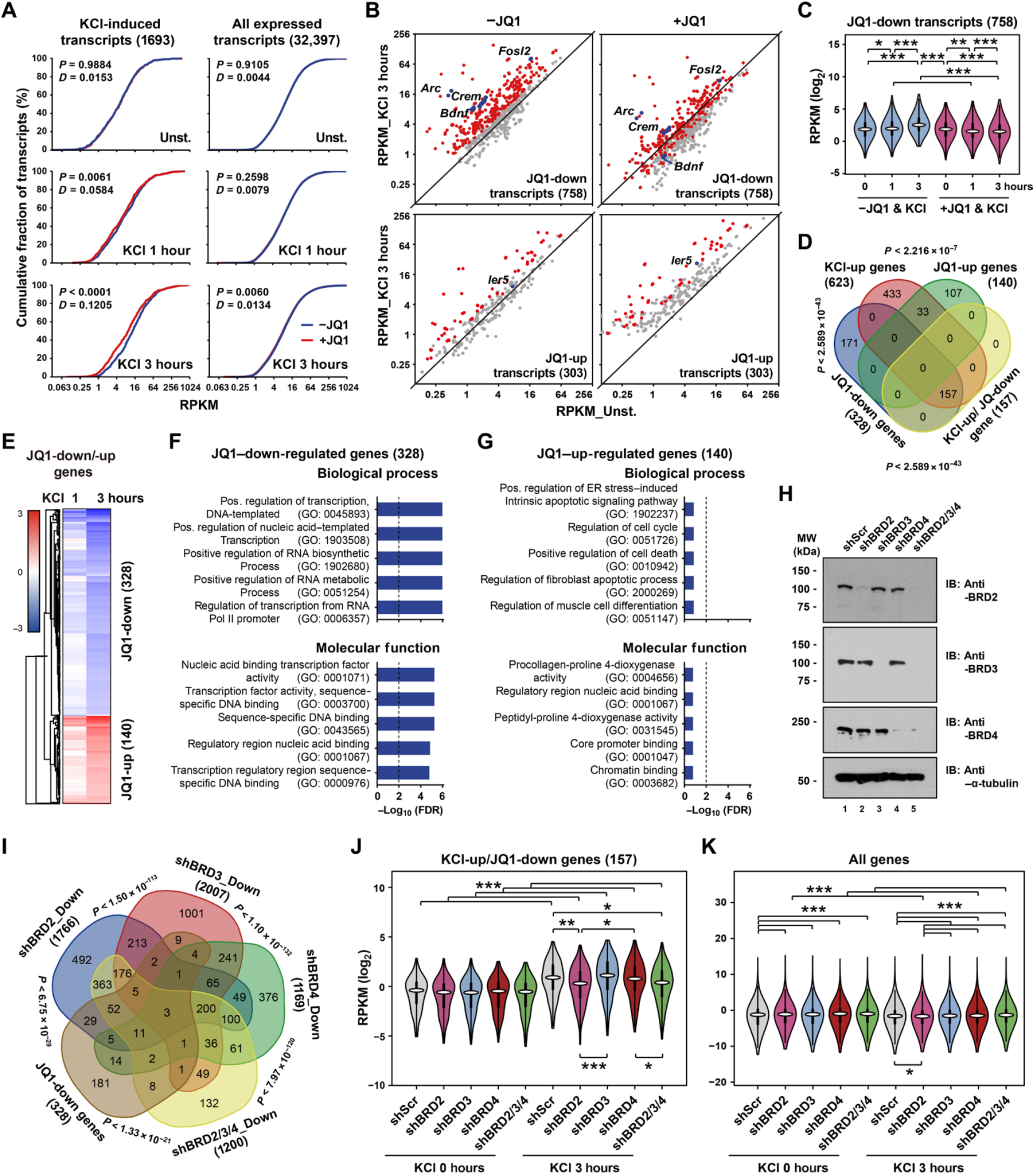
BET proteins are functionally important for activity-induced transcription. (**A**) Cumulative plots showing the differences in expression levels (RPKM) of KCl-induced and all expressed transcripts between +JQ1- and −JQ1-treated neurons. (*P* values were from Kolmogorov-Smirnov test). (**B**) Scatterplots showing the reads per kilobase per million mapped reads (RPKM) values of JQ1-down or JQ1-up regulated transcripts in unstimulated and KCl-depolarized neurons (all dots). Red dots, KCl–up-regulated transcripts; blue dots, the genes of our interest. (**C**) Violin plots showing RPKM (log_2_) changes of JQ1-down transcripts in different conditions. (*P* values were from two-tailed unpaired *t* test with Welch’s correction). (**D**) Venn diagram showing the overlaps between different gene groups. (**E**) Heatmap showing the expression FC of JQ1-down or JQ1-up genes at KCl depolarized over unstimulated condition. (**F** and **G**) Top five GO terms of JQ1-down (F) and JQ1-up genes (G). Dashed lines, *P* = 0.01. ER, endoplasmic reticulum. (**H**) Western blotting data showing the KD efficiency of BET proteins. MW, molecular weight; IB, immunoblotting. (**I**) Venn diagram showing the overlaps between the genes down-regulated by each shBET- and JQ1-down genes. (*P* values were from hypergeometric test). (**J** and **K**) Violin plots showing RPKM (log_2_) of KCl-up/JQ1-down (J) and all genes (K) in unstimulated or KCl-stimulated conditions. (*P* values were from two-tailed unpaired *t* test with Welch’s correction).

To assess the effect of BET inhibition during activity-induced gene expression, primary cultures of mouse cortical neurons were pretreated with either +JQ1 (500 nM; BET bromodomain inhibitor) or −JQ1 (500 nM; stereoisomer of +JQ1, which has no significant interaction with any BET) for 5 min and then membrane-depolarized by KCl to trigger activity-dependent transcription. mRNA sequencing (mRNA-seq) libraries were prepared from total RNAs collected at 1 and 3 hours following depolarization to examine the effect of JQ1 during the early stage of activity-induced transcription (Fig. 1A and fig. S1A). DESeq2 was used to identify differentially expressed genes (DEGs) between the conditions [+JQ1 versus −JQ1 at a matched time point (unstimulated, 1 hour, or 3 hours), fold change (FC) > 1.5, and false discovery rate (FDR) < 0.05]. The analysis found that 328 (758 transcripts) and 140 (303 transcripts) genes were reproducibly down-regulated (JQ1-down) and up-regulated (JQ1-up) by +JQ1 treatment, respectively (Fig. 1, A to C; fig. S1, B and C; and table S1). Other than these DEGs, acute BET inhibition had little impact on global transcriptome profiles of all expressed transcripts (Fig. 1A, right). About half of the JQ1–down-regulated genes were overlapped with KCl depolarization–induced genes (KCl-up), suggesting that BET proteins are functionally involved in activity-induced transcription (Fig. 1D and fig. S1D). Consistent with recently shown effect of JQ1 on fear memory (*33*), several learning and memory-related genes, such as *Arc*, *Fosl2*, *Crem*, and *Bdnf*, were included in the JQ1-down gene group (Fig. 1B and fig. S1B). Gene ontology (GO) analysis indicates that JQ1-down genes are significantly associated with gene transcription (Fig. 1, E and F). This result is consistent with the previously known fact that most immediate early genes (IEGs) encode transcription factors (TFs) that function in the subsequent wave of gene induction (*40*). JQ1-up genes show fewer overlaps with KCl-up genes (33 of 140 genes) (Fig. 1D) and no significant GO enrichment for any biological process or molecular function (Fig. 1G). Several memory-related genes present in JQ1-down or JQ1-up genes were further validated by real-time quantitative polymerase chain reaction (RT-qPCR) following JQ1 treatment in neurons (fig. S1E). Therefore, the primary function of BET proteins might be to promote activity-dependent gene expression.

To further validate that JQ1’s effect on gene expression is mediated by BET family proteins, we knocked down all three BET proteins and examined the transcriptome-wide changes by mRNA-seq (fig. S1F). A larger number of genes were affected by triple (BRD2/3/4) knockdown (KD) than JQ1. This could indicate that BET proteins function in transcription through both bromodomain-dependent and bromodomain-independent pathways as previously suggested (*9*–*11*). It could also reflect the difference in the duration of BET inhibition. JQ1 was added only 5 min before the KCl stimulation to minimize its secondary effect, whereas triple KD had to be performed for 3 days to ensure sufficient KD of all BET family proteins, during which any indirect changes in transcriptome could accumulate in addition to the changes occurred at direct BET protein target genes. Nonetheless, we observed that most of JQ1-down genes show a trend toward a decrease in transcription upon triple KD. Several memory-related genes (*Fosl2*, *Crem*, *Arc*, *Bdnf*, and *Nr4a2*) present in JQ1-down genes were further validated by RT-qPCR following the triple KD in neurons (fig. S1G). Therefore, JQ1-mediated changes in gene transcription most likely occur by alteration in BET protein functions.

We next examined the role of each BET family protein in activity-dependent gene transcription. BRD2/3/4 were known to be ubiquitously expressed (*2*, *12*, *21*), and we further confirmed their coexistence in the mouse cortex at a single-cell level by analyzing publicly available single-cell transcriptome data (fig. S2A and table S2). BRDT was known to be testis specific, and we also confirmed its extremely low RNA level in mouse cortical neurons (fig. S2B). We then performed short hairpin RNA (shRNA)–mediated KD of individual BET family proteins to examine their unique and/or redundant functions. In this experiment, we performed global run-on sequencing (GRO-seq) following each KD or triple KD (Fig. 1H) to gain a better sensitivity and accuracy in assessing the impact of BET protein KD in RNA polymerase II (Pol II) transcriptional activity. For the KCl-up or JQ1-down genes that we identified from mRNA-seq, KD of each BET family protein had differential effects in transcription (Fig. 1, I to K; fig. S2, C to F; and table S3). BRD2 KD caused the most significant decreases in transcription. Single KD of BRD3 and BRD4 had a little and moderate effect, respectively. The most marked effect of BRD2 single KD was unexpected since BRD4 dysregulation is widely believed to be responsible for JQ1-mediated inhibition (*14*–*16*). The overlap among individual KD-regulated genes (Fig. 1I and fig. S2C) further suggests that BET family proteins could work redundantly or distinctively depending on the context of the target genes.

### JQ1 differentially affects BET protein bindings during activity-dependent transcription

**Fig. 2:**
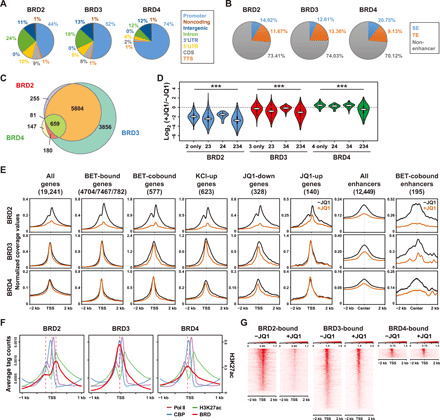
JQ1 differentially affects the recruitment of BET proteins. (**A**) Pie chart showing the genomic distribution of BET proteins. (30-min KCl-depolarized neurons). 3′UTR, 3′ untranslated region; 5′UTR, 5′ untranslated region; CDS, coding sequence; TTS, transcription termination site; BRD, bromodomain-containing protein. (**B**) Pie chart showing the fraction of BET protein master peaks present at enhancers and non-enhancers. (**C**) Proportional Venn diagram showing the overlap between each BET protein master peak. (**D**) Violin plots showing the FC of the ChIP-seq mater peaks (tag counts in +JQ1 over −JQ1) present in the gene body and up to 5-kb upstream regions of annotated genes. The genes were categorized on the basis of the co-occupancy of BET proteins. (*P* values were from Kruskal-Wallis test). (**E**) ChIP-seq read density plots of each BET protein at the transcription start sites (TSSs) or the enhancer centers of different gene categories. (−JQ1 and +JQ1 conditions). (**F**) ChIP-seq read density profiles of Pol II, H3K27ac, CBP, and each BET protein around the TSSs. (**G**) Heatmaps showing the ChIP-seq signals for each BET protein around the TSSs of BET-bound genes.

JQ1 has similar affinities to the bromodomains of BET family proteins in vitro (*4*, *12*). To examine the sensitivity of individual BET family proteins to JQ1 in an in vivo context, we analyzed the global binding profiles of BRD2/3/4 in neurons treated with or without JQ1 during activity-induced transcription. Chromatin immunoprecipitation (ChIP)–qPCR results showed that all three BET proteins are inducibly recruited to the promoters of several JQ1-down genes (*Fosl2*, *Crem*, and *Arc*) after depolarization, which was significantly blocked by JQ1 pretreatment (fig. S3A). JQ1 is a specific BET bromodomain inhibitor with no detectable binding to other bromodomain-containing proteins (e.g., CBP) in the range of dose used in most studies (*15*). To further examine this issue, we performed ChIP-qPCR analyses of CREB-binding protein (CBP) and E1A binding protein p300 (p300) and found no difference in their binding at several target promoters (fig. S3B). Therefore, ChIP sequencing (ChIP-seq) was carried out in neurons harvested at 30 min after depolarization (fig. S3, C and D). The peak annotation revealed that BRD2/3/4 are prominently enriched at the promoters and within the gene body (>86% of the total peaks) (Fig. 2A). A stark contrast was that a small portion (26 to 30%) of BET protein binding sites are located to the enhancer regions (Fig. 2, A and B). We also observed extensive overlaps between the binding sites of these factors, implying possible functional redundancies (Fig. 2C). To examine whether JQ1 sensitivity differs depending on the combination of overlapped peaks, we categorized the binding peaks of all three BET proteins based on their overlaps with one another (e.g., BRD2 peak alone, BRD2/3-cobound, BRD2/3/4-cobound, etc.) and then examined any differences in JQ1 sensitivity between the categories (Fig. 2D and fig. S4A). In all categories, BRD2 was more vulnerable to JQ1 than BRD3 and BRD4. BRD4 showed the least sensitivity to JQ1, and even more, about one-third of BRD4 peaks were actually increased by JQ1. We further observed that JQ1 sensitivity is generally correlated with the degree of overlaps among BET protein peaks. Those peaks overlapped by all three BET proteins were most sensitive to JQ1 (Fig. 2D). The peak annotation showed that BET proteins bind to the promoters and/or the coding regions of 11,214 genes. RNA sequencing (RNA-seq) analysis estimated that 12,723 genes are transcriptionally active, producing RNA reads higher than the background, and more than two-thirds of them were directly bound by BET proteins (fig. S4, B and C). Therefore, BET proteins bind to most of transcriptionally expressed genes in various combinations. Notably, the majority of BRD2 peaks coexist with BRD3 at the promoters (95.03% of total BRD2 peaks), suggesting their close relationship (Fig. 2C and fig. S4A). In contrast, BRD3 and BRD4 not only work together with the other family members but also regulate some genes independently of other family members.

The JQ1 sensitivity patterns at the promoters or enhancers of different gene categories were similar to those shown by the peak-based analysis (Fig. 2E). BRD2 binding levels were most markedly decreased by JQ1 across all categories, whereas BRD4 binding was least affected or even higher with JQ1 (Fig. 2E and fig. S4A). Despite the large number of genes occupied by BET family proteins, only 328 genes were significantly down-regulated by JQ1 (Figs. 1, B and E, and 2E). For these genes, all three BET members show significantly reduced binding at the promoters upon JQ1 treatment, suggesting that the bromodomain-dependent recruitment of BET proteins is critical for these selected genes. We also noted that the binding levels of BRD3 and BRD4 were relatively lower at the promoters of JQ1-down genes than those at other gene groups (Fig. 2E; note that *y*-axis values at different gene categories differ). Therefore, the prominent binding of BRD2 at this gene group that becomes more effectively inhibited by JQ1 might be, in part, responsible for selective transcriptional down-regulation. The binding levels of BET proteins at the promoters of JQ1-up genes are either slightly reduced (BRD2) or not reduced at all (BRD3/4) by JQ1 (Fig. 2E), suggesting that increased transcription by JQ1 at these genes is most likely due to a secondary effect. While different JQ1 sensitivity of individual BET family proteins suggests that each BET family member differentially uses the bromodomain-dependent function, other factors such as potential differences in the chromatin on-off rate might also contribute to the observed differences. Prolonged JQ1 treatment (overnight) significantly reduced all three BET proteins’ binding to the promoters where BRD2 was only affected by the short-term treatment (fig. S4, D and E).

Together with peak-based analysis, these results suggest that BRD2 recruitment to the chromatin depends heavily on its bromodomain-mediated interaction with acetylated proteins. In contrast, a large portion of BRD3 and BRD4 recruitment might be made through a bromodomain-independent mechanism (e.g., ET domain–mediated interactions with transcription regulators). This idea is further supported by the aggregate plots (Fig. 2F). The average density of BRD2 ChIP-seq reads surrounding the transcription start sites (TSSs) of all annotated genes exhibits a distinct bimodal shape that coincides with positioned nucleosomes flanking the TSSs determined by the peak densities of H3K27ac, which suggests that BRD2 is recruited to acetylated nucleosomes. In contrast, both BRD3 and BRD4 show a single average peak that is situated within the nucleosome-free regions (NFRs) where transcription complexes are assembled. Thus, various transcriptional regulators present in the NFRs most likely play a role in the recruitment of BRD3/4. Since the binding of most BRD3 and BRD4 was not affected by JQ1, bromodomain-independent recruitment might be a dominating mechanism in this case. These aggregated peak patterns could also be seen by heatmaps (Fig. 2G). This model is based on the average peak intensities at all annotated gene promoters, thus we also anticipate that a different mode of recruitment could occur for each BET protein depending on the gene context. Nonetheless, it is conceivable that BET protein recruitment to the target genes is a coordinated process involving both bromodomain-dependent and bromodomain-independent interactions. Together, the chromatin occupancy profiles support the findings from the transcriptome analysis that BET family proteins can function together or independently depending on the gene context. For shared target genes, individual BET proteins are recruited by distinctive mechanisms, during which BRD2 and BRD3 more closely coordinate with each other than with BRD4.

### Enhancer-bound BET proteins are more sensitive to JQ1

**Fig. 3:**
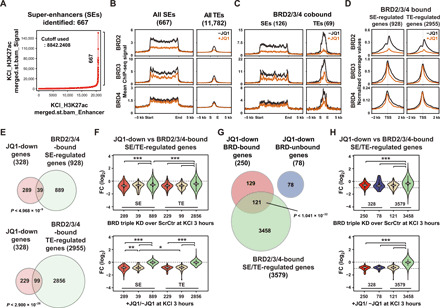
Comparison of BET protein binding profiles between SEs and TEs. (**A**) Ranking of super-enhancer (ROSE) analysis using H3K27ac ChIP-seq data that determines SEs. (**B** and **C**) ChIP-seq read density plots at all or BRD2/3/4-cobound SEs or TEs. (**D**) ChIP-seq read density plots of BET proteins near the TSSs of BET-bound genes that are located within ±50 kb from the SE or TE (SE/TE-regulated genes). (**E**) Venn diagram showing the overlap between JQ1-down and BRD2/3/4-bound SE/TE-regulated genes. (*P* values were from hypergeometric test). (**F**) Violin plots showing the RPKM-FC (log_2_) values of the genes in each category shown in (E) after BRD2/3/4 triple KD (top) or JQ1 treatment (bottom). (**G**) Venn diagram showing the overlaps among JQ1-down/BET-bound genes, JQ1-down/BRD-unbound genes and BRD2/3/4-bound SE/TE-regulated genes. (*P* values were from hypergeometric test). (**H**) Violin plots showing the RPKM-FC (log_2_) of the genes in each category shown in (G) after BRD2/3/4 triple KD (top) or JQ1 treatment (bottom). (*P* values were from Mann-Whitney *U* test).

Previous studies suggested that the transcriptional impact of BET inhibition occurs at a rather small set of genes (*2*, *3*). Such a selective effect was attributed to the preferential disruption of BRD4 binding at the enhancers (*9*, *27*). We next analyzed how JQ1 affected the binding of BRD2/3/4 at the enhancers. A total of 12,449 enhancers were identified on the basis of the H3K27ac-enriched peaks. We then split them into super-enhancer (SE; 667) and typical-enhancer (TE; 11,782) groups, as previously reported (Fig. 3A) (*9*). The average enrichment level of BET proteins at all SEs was much higher than that at all TEs (Fig. 3B). However, when the enhancers were further divided by the combinatorial binding patterns of BET family members, we found that TE-bound BET proteins show a higher enrichment than SE-bound BET proteins in all subgroups (Fig. 3C and fig. S4, F and G). We infer that in addition to SEs, those subsets of TEs strongly bound by BET proteins might be actively engaged in activity-induced transcriptional control. In all cases, the enhancer-bound BET proteins are more vulnerable to JQ1 treatment compared to the promoter-bound BET proteins, although BRD2 still showed the highest sensitivity to JQ1 among all BET family members (Figs. 2E and 3, B and C, and fig. S4, F and G). This observation is consistent with previous reports demonstrating that the bromodomain-dependent recruitment of BET proteins is a prominent feature of the enhancers (*9*).

We next examined how many of the JQ1-down genes resulted from reduced BET protein activity at the enhancers. We identified 928 and 2955 genes as SE- and TE-regulated genes, respectively, based on the ±50-kb window centered on BET-bound enhancers (Fig. 3, D and E, and fig. S4, H to J). GO analysis indicates that BET-bound SEs are significantly associated with gene transcription activities, neuron related or differentiation/development related, and TE-regulated genes are significantly associated with gene transcription activities, RNA processing, or protein modification (table S4). About one-third of the JQ1-down genes (121 of 328 genes) belong to the enhancer-regulated genes. We did not see any bias in the overlap of JQ1-down genes with the SE- or TE-regulated gene group (Fig. 3E and fig. S4J), suggesting that both SE- and TE-associated BET proteins contribute to the regulation of target genes. All of those enhancer-associated and JQ1-down genes also showed significant levels of BET protein binding at their promoters (Figs. 2E and 3D and fig. S4, H and I). One hundred twenty-nine genes (39.3%) of the remaining JQ1-down genes had BET protein bindings only at the promoter regions, leaving only 78 genes (23.7% of JQ1-down genes) as BET protein independent (Fig. 3G and fig. S4L). Therefore, about two-thirds of JQ1-down genes are considered as direct targets of BET proteins acting on the enhancers and/or the promoters. We next examined transcriptional changes associated with different enhancer subgroups. As expected, a triple KD of BRD2/3/4 led to a significant decrease in transcription of JQ1-down genes (Fig. 3F and fig. S4K). Although only a small portion of the enhancer-regulated genes (121 of 3579) was JQ1-down, the remaining enhancer-regulated genes (3458 genes of 3579 genes) became significantly down-regulated by a triple KD of BRD2/3/4 regardless of their associations with SEs or TEs (Fig. 3H and fig. S4M). This pattern of transcriptional effect was also observed when the enhancers were separately analyzed for the SE and TE groups (Fig. 3F and fig. S4K). Given that the enhancer-bound BET proteins are significantly disrupted by JQ1 treatment, those enhancer-regulated genes that are insensitive to JQ1 treatment (Fig. 3, F and H) could indicate that promoter-associated BET proteins in this category function through bromodomain-independent mechanisms. Together, our dissection defines that BET proteins regulate transcription through their combinatorial actions at selected promoters and enhancers without any biased usage between SEs and TEs. Although more sensitive perturbation of BET function at the selected enhancers could account for JQ1-mediated transcriptional down-regulation, a similar portion of JQ1-down genes showed promoter-dependent activity of BET proteins.

### Sequential recruitment of BET proteins

**Fig. 4:**
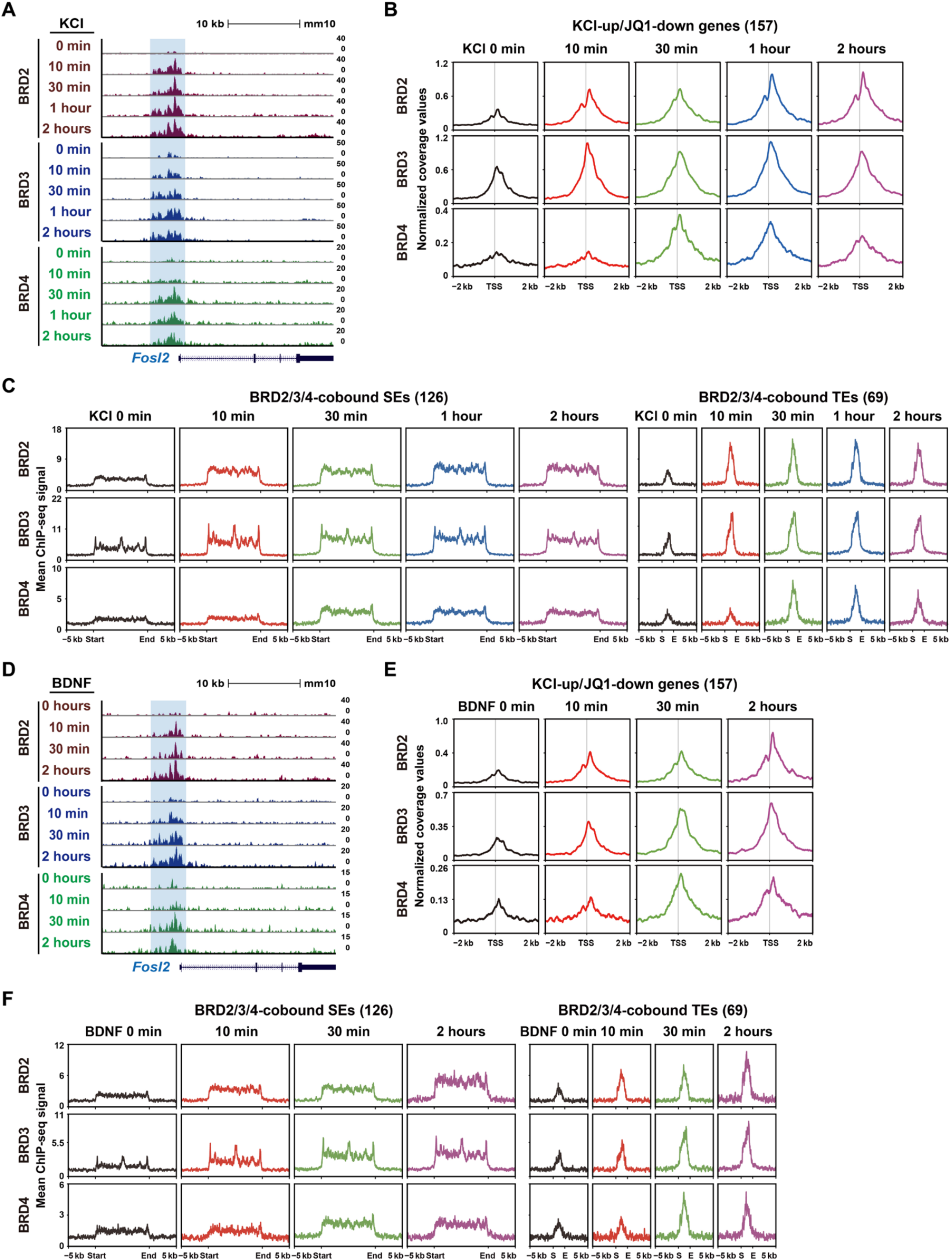
BET proteins are recruited to the cis-regulatory regions with different kinetics. (**A**) ChIP-seq tracks showing the binding kinetics of each BET protein at the *Fosl2* gene locus after KCl depolarization. (**B**) The average ChIP-seq signals of BET proteins at different time points following KCl depolarization are shown at the TSS regions of KCl-up/JQ1-down genes. (**C**) The average ChIP-seq signals at BRD2/3/4-cobound SEs or TEs following KCl depolarization. (**D**) ChIP-seq tracks showing the binding kinetics of each BET protein at the *Fosl2* gene locus after BDNF stimulation (10 ng/ml). (**E**) The average ChIP-seq signals of BET proteins at different time points following BDNF stimulation are shown at the TSS regions of KCl-up/JQ1-down genes. (**F**) The average ChIP-seq signals at BRD2/3/4-cobound SEs or TEs following BDNF stimulation.

To examine the mechanism of BET protein coordination, we next investigated the recruitment kinetics for each family member by performing ChIP-seq in neurons at different time points following membrane depolarization. Neuronal activity was first suppressed by tetrodotoxin (TTX; quiescent neurons; unstimulated; 0 min); then, neurons were synchronously depolarized for various durations (10, 30, 60, and 120 min of KCl treatment). All BET members show strong activity-induced binding at cis-regulatory regions consistent with BET protein involvement in activity-dependent gene expression (Fig. 4, A to C, and fig. S5, A to C). Induced binding of BRD2 and BRD3 occurred immediately after the activity increase (10 min), followed by BRD4 binding at 30 min. Notable was that unlike the other members, a substantial level of BRD3 binding was present in quiescent neurons (0 min), with more BRD3 recruited together with BRD2 immediately following the activity increase. Binding of BRD2 and BRD3 persists until the measured duration (2 hours), whereas BRD4 begins to decline after 30 min of peak, showing a more dynamic induction pattern. These results suggest a temporally differentiated action of each BET member during activity-induced transcription in neurons. This pattern of distinctive recruitments also occurs in response to a different stimulus, brain-derived neurotrophic factor (BDNF), which induces genes that are crucial for neuronal function and survival (*35*). Similar to the KCl-induced responses, BET proteins showed distinct temporal binding patterns upon BDNF treatment (Fig. 4, D to F, and fig. S5, D to F). But in this case, the level of prebound BRD3 before BDNF treatment was lower than that in the TTX-mediated quiescent condition in membrane depolarization experiments (compare Fig. 4, B and E, at 0 min). The difference might be due to ongoing spontaneous neuronal firing activity in the BDNF experiment that prevents the stable association of BRD3 before BDNF addition. Temporally defined binding patterns of BET proteins are consistent with the model that BET family proteins functionally coordinate with each other during transcriptional activation. In light of the transcriptional activation process, the observed sequential recruitment could imply the distinctive roles of individual BET family members in gene activation. For example, BRD3 might have a role in the suppression of activity-dependent genes before an activation signal and then becomes a part of the activator complex together with BRD2 that functions to initiate the transcriptional activation process. Delayed association of BRD4 suggests its role in a later stage of transcription such as Pol II elongation (*7*, *9*), as the BRD4-binding peaks coincide with the first wave of IEG transcription.

### Chromatin recruitment of BRD2 and BRD3 occurs in an interdependent manner

**Fig. 5:**
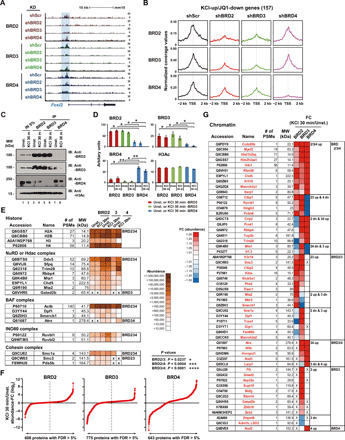
Recruitment of BRD2 and BRD3 is interdependent. (**A**) Representative ChIP-seq tracks at the *Fosl2* gene locus. ChIP-seq was performed in neurons after KD of each BET family member or Scrambled control (Scr). (**B**) The average occupancy profiles of BET proteins around the TSSs of KCl-up/JQ1-down genes after KD of each BET protein. (**C**) Co-IP experiment with nuclear extracts prepared from unstimulated or KCl-depolarized neurons. (**D**) Quantification of co-IP Western blot using ImageJ. Bar graphs show means ± SEM. (*P* values were from two-tailed unpaired *t* test with Welch’s correction). (**E**) BET interacting proteins determined by RIME in histones and chromatin remodeling complex categories. (**F**) The FC in abundance (log_2_) of BRD2-, BRD3-, or BRD4-associated proteins between unstimulated and KCl-stimulated conditions. The proteins are ranked by the log_2_ FC values (KCl 30 min/Unst.). (*P* values were from Mann-Whitney *U* test). (**G**) BET-associated proteins with chromatin-related functions. The genes are grouped on the basis of the direction of abundance changes in each BET protein complex. The color scale indicates the FC of abundance (KCl 30 min/Unst.). (*P* values were from two-tailed unpaired *t* test with Welch’s correction).

The recruitment kinetics analysis described above indicates that BRD2 and BRD3 might work closely with one another, especially during the early stage of transcriptional activation. To gain additional molecular insight into the coordination between BET family members, we performed a single KD of each family member and examined its impact on chromatin occupancy of the other family members. KD of either BRD2 or BRD3 alone markedly decreased the chromatin occupancy of the other (Fig. 5, A and B, and fig. S6A). The BRD4 binding level was also reduced but to a lesser degree. BRD4 KD did not affect the binding levels of BRD2 and BRD3. This result indicates that BRD2 and BRD3 coordinate with each other for their recruitment to the cis-regulatory regions. Consistently, co-immunoprecipitation (co-IP) assay using nuclear lysates prepared from neurons with and without membrane depolarization showed that BRD2 and BRD3, but not BRD4, were efficiently pulled down by reciprocal IPs (Fig. 5, C and D). We next analyzed protein complexes associated with each BET family member by rapid immunoprecipitation mass spectrometry of endogenous proteins (RIME), which is a formaldehyde cross-linking–based method that analyzes endogenous protein complexes, especially chromatin and TF complexes by mass spectrometry (MS) (*41*). A total of 781 proteins were identified as BET-associated proteins (FDR < 0.05). As expected for the epigenetic reader, all histone subunits were significantly enriched in the BET-immunoprecipitated fractions (Fig. 5E). In addition, BET complexes contain a number of chromatin-associated proteins that were previously shown to interact with BET proteins such as barrier-to-autointegration factor (BAF) and nucleosome remodeling and deacetylase (NuRD) complexes (*8*, *22*, *24*, *42*, *43*). The majority of the BET-associated proteins were commonly identified in individual family members’ IP fractions, indicating that all three family members jointly participate in the formation of protein complexes. However, each BET family member exhibits rather distinctive dynamic ranges of interactions with associated proteins in an activity-dependent manner (Fig. 5F and fig. S6, B to D). BRD4 showed the highest dynamic range of interactions with associated proteins whereas BRD2-associated complexes were relatively stable with activity change. GO analysis revealed that a significant portion of BET-interacting proteins functions in RNA processing, translation, and metabolism (table S5, A to C), suggesting broad functions of BET proteins beyond the regulation of chromatin and transcription. We then selected the proteins with chromatin- and transcription-related functions and examined their association patterns with each BET family member in response to KCl depolarization (Fig. 5G, fig. S6E, and table S5, D to K). Most of the proteins were associated with more than one BET family members, although neuronal activity alters their association with each BET member differently. BRD3 appears to be the common component in most of the protein complexes in these categories. These results demonstrate that the chromatin regulation is highly coordinated by all three BET family members with distinctive functional contributions in an activity-dependent manner. Together with the kinetics analysis (Fig. 4), we suggest a mechanistic model that upon KCl depolarization, BRD2 and BRD3 are rapidly recruited to the chromatin in an interdependent manner. The subsequent binding of BRD4 completes the formation of the functional transcription complexes required for productive transcriptional induction. In this regard, individual BET proteins might function at different stages of transcription such that the main role of BRD2/3 might be to remodel the promoters and enhancers to activate the transcriptional initiation process, whereas BRD4 is primarily involved in transcription elongation. This model is consistent with previously shown BRD4 function in transcription elongation (*7*, *9*).

### Individual BET proteins differentially affect memory formation

**Fig. 6:**
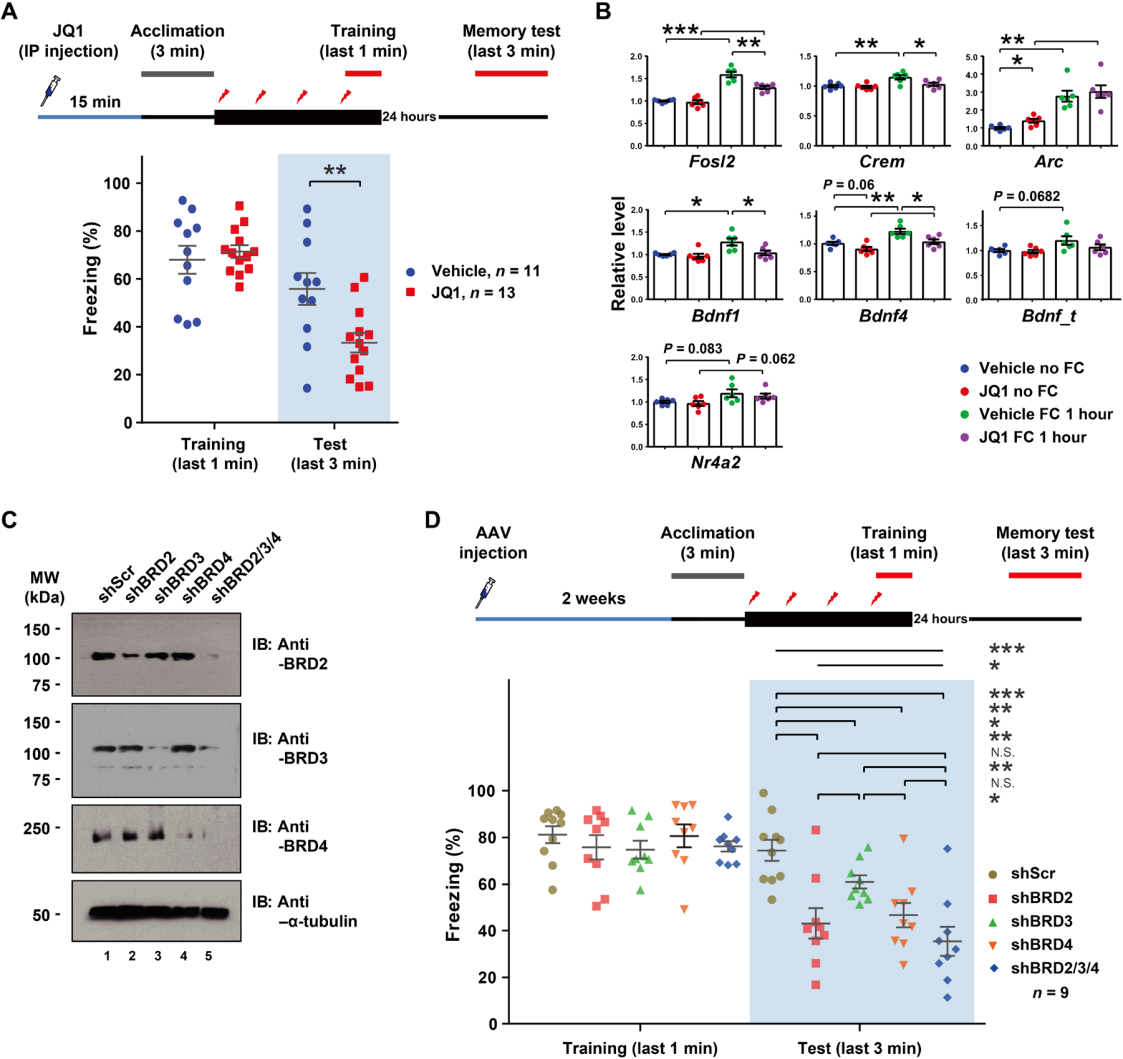
JQ1 and KD of BET family proteins impair LTM. (**A**) Long-term fear memory test. Schematics of conditioning procedure (top) and freezing behavior results during the initial training and test period are shown (bottom, *n* = 11 or 13 for vehicle or JQ1). (**B**) RT-qPCR data from cortical tissues extracted from mice treated as indicated (*n* = 6). Three different alternative transcripts of the *Bdnf* gene were included (*Bdnf*_t: common exon, *Bdnf*_1: exon 1, *Bdnf*_4: exon 4). Bar graphs show means ± SEM. (*P* values were from two-tailed unpaired *t* test with Welch’s correction). (**C**) Western blotting of each BET protein from the hippocampal tissues extracted from AAV-infected regions. (**D**) Long-term fear memory test with mice treated with Scr, single, or triple KD of BET proteins (*n* = 9). (*P* values were from one-way analysis of variance with Tukey’s posttest). N.S., not significant.

BET inhibition was shown to improve pathological conditions of autism and neurodegeneration by altering gene expression programs in the brain (*35*–*37*, *39*). Consistently, our JQ1-down gene group also includes several learning and memory-related genes (e.g., *Fosl2* and *Bdnf*) (Fig. 1B). We next examined the functional involvement of each BET family protein in the formation of LTM, which has not been demonstrated in previous studies (*33*, *34*). We first performed a contextual fear memory test with JQ1 or vehicle (dimethyl sulfoxide) injected 15 min before conditioning to determine the effect of an acute BET inhibition in LTM. Because of the acute immobility effect of +JQ1 at high doses, we optimized the dose (12.5 mg/kg) that does not affect the mobility of mice (fig. S7A). Under this condition, JQ1-injected mice exhibited a level of freezing similar to that of the vehicle-treated mice during fear conditioning. However, their freezing rates upon reexposure to the context in the following day (24 hours) were significantly lower than the vehicle control group, indicating impairment in LTM consolidation (Fig. 6A). Since JQ1 was injected only once at 15 min before contextual conditioning with a short half-life (~1 hour), this result suggests that BET family proteins play a role in memory formation by participating in the rapid induction of gene expression during the early stage of memory formation in vivo. We also measured RNA levels of several memory genes in the cortex and hippocampus isolated from fear-conditioned mice with and without JQ1 treatment and found that JQ1 inhibited induction of *Fosl2*, *Crem*, and *Bdnf* (isoforms 1 and 4) in at least one brain region (Fig. 6B and fig. S7B). Therefore, the behavioral impairment caused by JQ1 is associated with transcriptional changes in some of the key memory genes in vivo.

We next examined the functional contributions of individual BET family members in memory formation in vivo. We stereotaxically injected AAVs (adeno-associated viruses)–expressing shRNAs against each BET family members or all three members into the hippocampus. Two weeks later, AAV-injected mice were subjected to contextual fear conditioning. The KD efficiency was validated at the protein level in the hippocampal tissues isolated from individual KDs (Fig. 6C). Triple KD of BET proteins showed the biggest effect in LTM (Fig. 6D and fig. S7C). However, KD of each BET family member alone was also sufficient to impair LTM albeit all weaker than the triple KD. The extent of LTM impairment caused by each KD was different such that BRD2 KD caused the most severe impairment, followed by KD of BRD4 and then BRD3. The observed differential impact in LTM mirrors the extent of gene expression changes caused by KD of each BET family members in neurons (Fig. 1J). These results collectively demonstrate that each BET protein differentially contributes to LTM by regulating activity-dependent gene expression.

### BET proteins are differentially impaired in the FXS

**Fig. 7:**
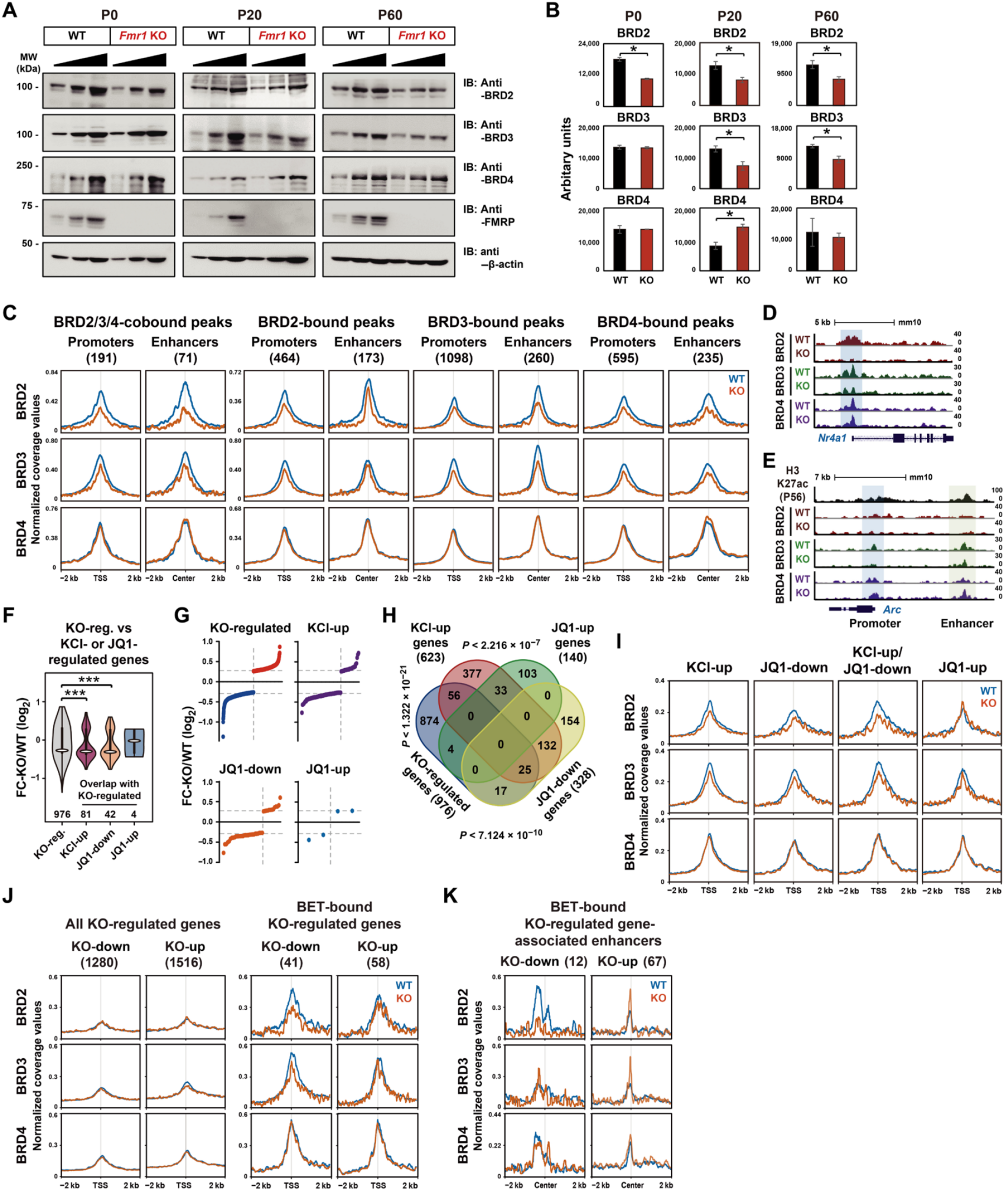
Expression and recruitment of BET proteins to the cis-regulatory regions are differentially altered in *Fmr1* KO. (**A**) Western blotting data showing representative expression data of BET and FMRP proteins in WT and KO cortices at P0, P20, or P60 that was from biological replicates (*n* = 2, 4, or 4). (**B**) The quantified average band intensities of three gradient lanes in each WT or KO in (A) using ImageJ were used for the graph presentation. Bar graphs show means ± SEM. (*P* values were from two-tailed unpaired *t* test with Welch’s correction). (**C**) ChIP-seq read densities of BET proteins in different categories at the cis-regulatory regions. (**D** and **E**) Representative ChIP-seq tracks at the *Nr4a1* and *Arc* gene loci. (**F**) Violin plots showing the RPKM-FC (log_2_) distributions of different categories of genes overlapped with KO-regulated genes. (*P* values were from Mann-Whitney *U* test). (**G**) The RPKM-FC (log_2_) in (F) are ranked in increasing order. (**H**) Venn diagram showing the overlap among different categories of genes. (*P* values were from hypergeometric tests). (**I**) ChIP-seq read densities of BET proteins around the TSSs of genes in different categories. (**J**) ChIP-seq read densities of BET proteins around the TSSs of KO-regulated genes in different categories. (**K**) ChIP-seq read densities of BET proteins centered on enhancers associated with BET-bound KO-regulated genes.

A previous study demonstrated that an *Fmr1*-encoded protein (FMRP) deficiency–caused increase in BRD4 protein level underlies the pathogenesis of FXS, and JQ1-mediated inhibition of BRD4 alleviates several phenotypes associated with FXS (*37*). To gain additional molecular insight into the BET protein function in FXS, we investigated all three BET proteins in *Fmr1* KO mice. We first compared the protein expression levels of BRD2, BRD3, and BRD4 in the cortices isolated from WT littermate controls and *Fmr1* KO at postnatal day (P) 0, 20, and 60. Expression of all three BET proteins was gradually decreased in WT (fig. S8A). Consistent with a previous report (*37*), the level of BRD4 protein was elevated in *Fmr1* KO compared to WT, but the level of the difference between WT and *Fmr1* KO varied with the developmental stage such that only P20 showed a significant difference (Fig. 7A). Unexpectedly, we also found that BRD2/3 expression was significantly decreased in *Fmr1* KO at P20 and P60 (Fig. 7, A and B, and fig. S8B). These changes in protein expression levels appear to occur at the translation level, as no significant changes in mRNA levels were detected in the previous report (*37*). We then performed ChIP-seq for each BET family member in cortical tissues isolated at P60 to see whether such changes in expression also affected the chromatin occupancy in *Fmr1* KO mice. The binding levels of both BRD2 and BRD3 at the cis-regulatory regions were significantly lowered in *Fmr1* KO mice, but BRD4 recruitment level was largely unchanged except at the BRD4-bound enhancers where BRD4 binding was marginally increased (Fig. 7, C to E, and fig. S8, C and D). These results could be confounded by heterogeneous cell types present in the brain, potentially masking the specific effects of *Fmr1* KO in neurons. To clarify this issue, we also analyzed fluorescence-activated cell sorting (FACS)–sorted neuronal nuclei from the cortices and observed the same effect of *Fmr1* KO in the expression of individual BET family proteins (fig. S8E).

To examine whether the decrease in the chromatin occupancy of BRD2 and BRD3 also contributes to the abnormalities of FXS, we analyzed mRNA-seq data generated from *Fmr1* KO neurons (*37*). FMRP deficiency induces up- and down-regulation of many genes (KO-regulated genes; FC > 1.2) with a trend toward more significant down-regulation (Fig. 7F). We compared this gene list with BET protein–regulated genes in two categories that required BET protein function, KCl-up and JQ1-down genes identified from our mRNA-seq in the same neuronal culture. The KCl-up genes that were also present in the KO-regulated group largely showed a decrease in transcription in *Fmr1* KO neurons (Fig. 7, F to H). Likewise, transcription levels of the JQ1-down genes were mostly decreased in *Fmr1* KO. The chromatin occupancy of BRD2 and BRD3, but not BRD4, was also significantly decreased at the promoters of the genes in these two groups (Fig. 7I). Although the comparison between the chromatin occupancy changes and DEGs was made with the data obtained from different experimental conditions (primary neuronal culture for DEG and P60 cortex tissue for ChIP-seq), these results are consistent with the model that perturbation of the BET protein recruitment might contribute to the transcriptional abnormality in FXS. We also examined the changes in peak intensity at the cis-regulatory regions of the KO-regulated genes to see whether there was a direct correlation between the BET chromatin occupancy and transcriptional changes in *Fmr1* KO (Fig. 7, J and K). Both BRD2 and BRD3 showed decreased binding at the promoters of KO–down-regulated (KO-down) genes but not at the KO–up-regulated (KO-up) genes. There was no change in BRD4 binding levels in either group. In the case of the BET-bound enhancers located within the ±50 kb from the KO-regulated genes, we observed decreased BRD2 binding at the enhancers associated with the KO-down genes (Fig. 7K). The enhancers associated with KO-up genes showed increased binding of all BET family members, especially BRD2 and BRD3. However, the fractions of KO-regulated genes that are directly bound by BET proteins (BET-bound KO-regulated genes) are quite small (Fig. 7J), indicating that the majority of transcriptional changes in *Fmr1* KO result from either indirect effects or posttranscriptional mechanisms. This is consistent with the well-established role of FMRP in translational control. As FXS is a neurodevelopmental disorder with deficits emerging early in development, it is also possible that the causative molecular changes could have occurred earlier than the age we investigated (P60). For example, P20 mice showed changes in all three BET family members (BRD2/3 down and BRD4 up) (Fig. 7A). It is possible that alterations in the expression of all three BET families collectively contribute to the FXS phenotypes. The KO-regulated gene group contains a number of histone modifiers and remodelers, which could collectively alter the chromatin landscape in *Fmr1* KO mice and influence BET recruitment (fig. S8F). Together, our analysis of the KO-regulated genes that are directly bound and regulated by BET proteins demonstrates that perturbation of the BET protein recruitment contributes to the transcriptional abnormality in FXS. Our study further implicates that JQ1 might not be an optimal solution for rescuing FXS, as the recruitment of individual BET family proteins is differentially altered in *Fmr1* KO mice.

### Inhibition of CBP/p300 HAT activity could reverse altered BET binding and rescue memory deficit of *Fmr1* KO mice

**Fig. 8:**
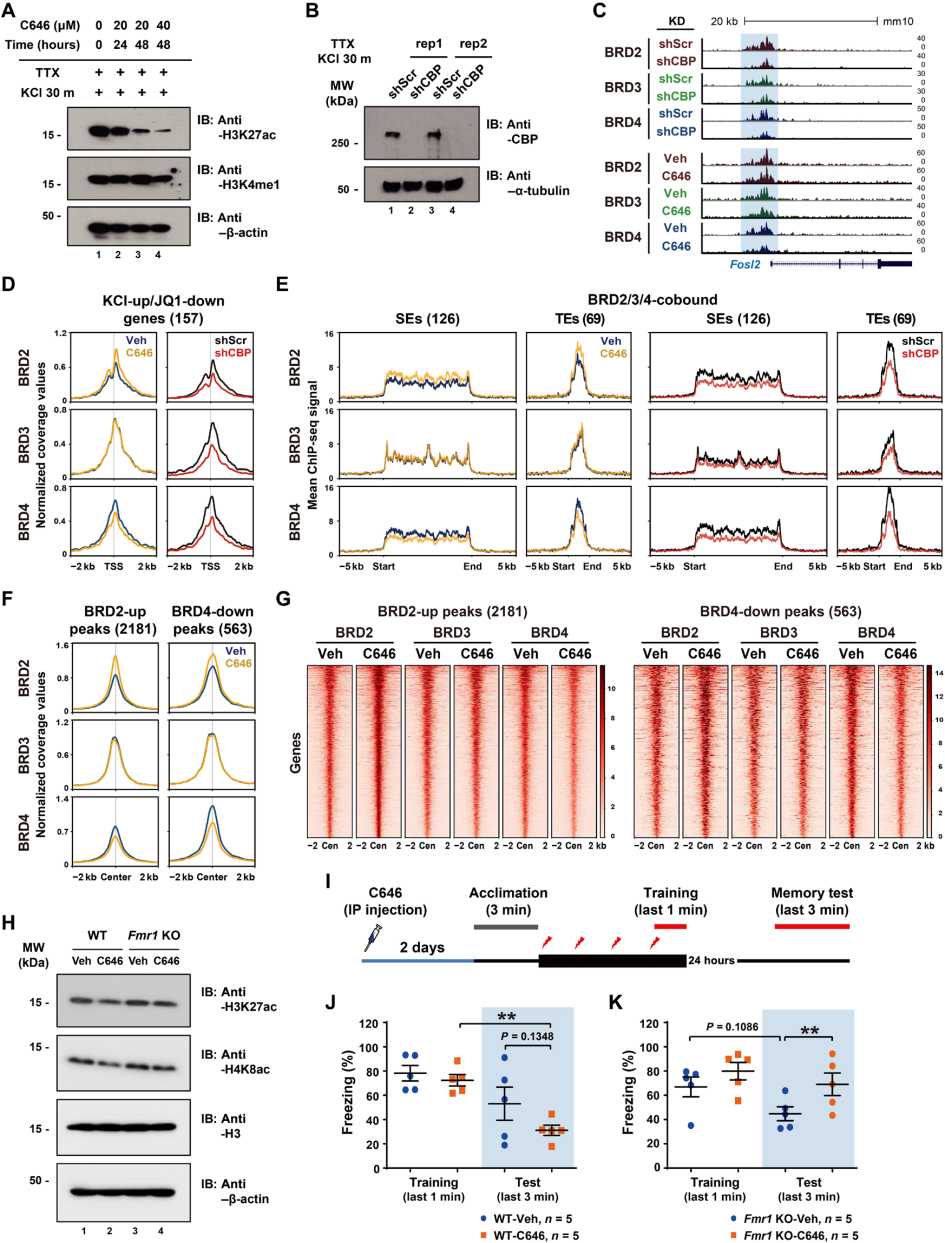
CBP/p300 HAT activity differentially affects BET protein recruitment, and its inhibition rescues the memory deficit of *Fmr1* KO. (**A**) Effect of CBP/p300 HAT inhibition by C646 in H3K27ac level in neurons. Quiescent neurons in TTX were added with C646, followed by KCl depolarization, and then subject to Western blotting. (**B**) The efficiency of lentivirus-mediated CBP KD. (**C**) Representative ChIP-seq tracks showing the *Fosl2* gene locus. C646 (20 μM) were treated for 48 hours. (**D**) ChIP-seq read densities of BET proteins around the TSSs of KCl-up/JQ1-down genes in two different paired conditions. (**E**) ChIP-seq read densities of BET proteins centered on BRD2/3/4-cobound SEs or TEs. (**F**) ChIP-seq read densities of BET proteins aligned at the centers of increased (BRD2-up) and decreased (BRD4-down) peaks by C646. (**G**) Heatmaps showing the intensities of BRD2-up and BRD4-down peaks in different conditions. (**H**) Effect of C646 injection in *Fmr1* KO mice. Whole-cell extracts from WT littermate and KO cortical tissues given vehicle or C646 (15 mg/kg) through intraperitoneal injection for 2 days were analyzed by Western blotting using the antibodies indicated on the right. (**I**) Scheme of experiment designs. (**J** and **K**) Effect of C646 in fear memory. WT or KO mice were given vehicle or C646 through intraperitoneal injection for 2 days (*n* = 5 each). *P* values were determined by two-tailed paired *t* test.

CBP/p300 HAT activity was known to be important for the bromodomain-dependent BRD4 recruitment to the chromatin (*20*). Having observed a range of JQ1 sensitivity in neurons, we next examined the role of CBP/p300 in the chromatin binding of individual BET family proteins. CBP/p300 HAT activity provides the substrates for BET protein binding by acetylating the lysine residues present at the histone tails as well as nonhistone proteins. We initially thought that BRD2 might critically rely on CBP/p300 HAT activity due to more prominent bimodal enrichment patterns flanking the TSSs that coincide with positioned nucleosomes and a higher sensitivity to JQ1 than the other BET family members. Unexpectedly, an acute inhibition of CBP/p300 HAT activity by a specific inhibitor C646 (20 μM) enhanced BRD2 recruitment to the cis-regulatory regions (Fig. 8, A and C to E, and fig. S9, A to D). No change was observed in BRD3 binding, and BRD4 was the only BET family member that showed decreased binding by C646. Increased BRD2 binding occurred at the regions where BRD4 binding levels were decreased, suggesting that loss of BRD4 binding was compensated by BRD2 (Fig. 8, F and G, and fig. S9E). This result illustrates that BET family members functionally coordinate with each other rather than simply acting in a redundant manner. Besides its HAT activity, CBP/p300 also functions as a coactivator to mediate protein-protein interactions with various types of transcriptional regulators. To see whether BET proteins require CBP/p300 coactivator function for their recruitment, we also performed CBP KD (Fig. 8B). In this case, all BET proteins showed decreased binding levels at the promoters and the enhancers, suggesting that a physical association with CBP also played a role in the BET recruitment to the cis-regulatory regions (Fig. 8, C to E, and fig. S9, A to D). Considering the average binding peak of BRD3 present within the NFRs as well as its insensitivity to JQ1 (Fig. 2, E and F, and fig. S4A), it might be that BRD3 is primarily recruited to their target loci through protein-protein interactions with CBP/p300 and other factors (Fig. 5G).

We noted that the altered binding patterns of BRD2 and BRD4 caused by C646 was opposite to those in *Fmr1* KO mice, which raised a possibility that CBP/p300 HAT inhibition by C646 might be able to alleviate the abnormality of *Fmr1* KO. Although C646 is not expected to reverse the abnormal decrease in BRD3 binding at the cis-regulatory regions, the contribution of BRD3 to gene expression and memory behavior was minimal in our analysis. C646 treatment for 48 hours substantially reduced the levels of H3K27ac and H4K8ac in both WT and *Fmr1* KO mice at P60, suggesting that these lysine residues are subject to CBP/P300-dependent acetylation (Fig. 8H and fig. S9F). Using this condition, we compared the effects of C646 in long-term fear memory between WT and KO mice. While C646-mediated inhibition of CBP/p300 HAT activity impaired LTM formation of WT, the memory deficit caused by *Fmr1* KO was effectively rescued to the degree shown by WT without any treatment (Fig. 8, I to K, and fig. S9, G and H). This result supports our conclusion that altered BET coordination could underlie the pathogenesis of FXS.

## DISCUSSION

BET family proteins have been studied in a wide range of cell types and diseases as key epigenetic regulators that control gene expression, and small-molecule inhibition of their bromodomains is recognized as an effective treatment option. Understanding the precise molecular mechanism of BET inhibition in diverse cellular contexts would be highly valuable not only for advancing our knowledge in epigenetic regulation but also for developing the treatment option suitable for each disease type. Our study demonstrated that individual BET proteins are recruited to the cis-regulatory regions with a hierarchical coordination. The chromatin binding profile in response to the inhibition of the BET bromodomain and CBP/p300 HAT activity was widely varied among the family members, suggesting that the recruitment of each BET family member was mediated by distinctive mechanisms. Our study with a mouse model of FXS further demonstrated that perturbation of BET protein coordination is, in part, responsible for altered transcription and resulting in the pathological conditions manifested by *Fmr1* KO.

Most BET inhibition studies attribute the effects of small-molecule inhibitors to dysregulation of BRD4 function, despite that currently available BET inhibitors exhibit similar affinities to the bromodomains present in all family members in vitro. Such a notion is due to either lack of studies with all major BET family proteins or, in some cases, the effects of pharmacologic BET inhibition were sufficiently recapitulated by BRD4 single KD (*33*). We confirmed that the three major BET family members, BRD2, BRD3, and BRD4, are quite ubiquitously expressed in the brain even at a single-cell level (fig. S2A and table S2) and, therefore, wondered whether JQ1 would affect all major family members equally or differentially in vivo. Although BET proteins extensively co-occupied the cis-regulatory regions, each BET protein differentially responded to JQ1 treatment in neurons with BRD2 being the most sensitive member (Figs. 2 and 3). To our surprise, BRD4 was marginally affected by short-term JQ1. The average occupancy peak of BRD2 near the TSS coincides with positioned nucleosomes flanking the cis-regulatory regions, whereas BRD3 and BRD4 were more enriched within the NFR. Such binding profiles could be relevant to the observed differential JQ1 sensitivity such that BRD2 primarily relies on acetylated histones for its recruitment to the cis-regulatory regions, which is effectively disrupted by JQ1. The interaction with various TFs located at the NFRs might be a preferential mechanism used by BRD3 and BRD4 with a varying degree of dependence on acetylation. The ET domain commonly present in BET family proteins could be an important player for this type of interactions to confer a binding specificity (*8*, *32*, *44*). Together, these results demonstrate that BET family members are recruited to their common target genes by distinctive mechanisms.

Our experimental system permitted an investigation of the coordination between the family members by monitoring BET recruitment and the effect of BET inhibition during the initial stage of transcriptional induction. Stimulus-induced gene expression occurs in a transcriptional wave over time (*45*). Rapid induction of IEGs by sensory stimulation is the first step in the transcriptional cascade, which has been shown to be critical for gene expression–dependent neuronal plasticity. We applied JQ1 to primary cultures of mouse cortical neurons 5 min before KCl-mediated gene induction. Likewise, our in vivo experiments were performed with an acute BET inhibition in which JQ1 was injected only 15 min before fear conditioning. Acute BET inhibition might have affected relatively a small number of genes, but it should help reveal a direct effect of BET inhibition without having secondary effects caused by a long-term inhibition. Our findings from the analysis of recruitment kinetics and protein-protein interactions (Figs. 3 and 4) suggest a model where BRD2/3 work together in the early stage of the transcriptional process such as promoter remodeling and initiation, whereas BRD4 primarily functions in a later stage such as transcription elongation (fig. S10). We also noted that a substantial fraction of BRD3 remains bound to the cis-regulatory regions even after a long period of activity suppression (Fig. 4), suggesting a possibility of a unique BRD3 function in gene repression. Our model integrates the coordination of all major BET proteins during transcriptional activation, which is consistent with previously reported features of individual family members. For example, BRD4 was shown to control transcriptional elongation by interacting with Mediator and P-TEFb (*1*, *6*, *8*, *13*, *46*). BRD2 was shown to be present in the complex with Pol II and TBP-associated factors, HATs CBP/p300, and chromatin assembly and remodeling factors (*29*, *42*). BRD3 is recruited by GATA binding protein 1 (GATA1) to both active and repressed genes in erythroblast (*47*).

An intriguing feature of BET inhibition is that, despite the widespread chromatin binding of BET proteins across a large number of genes and regulatory regions, only a selective gene set is affected by BET inhibition, which often occurs in a context or cell type–specific manner (*3*, *4*). Such selective effects of BET inhibition have been ascribed to the fact that BET proteins at enhancers are more sensitive to BET inhibitors (*9*, *27*). In particular, BET proteins bound at SEs were shown to be more sensitive to BET inhibition than TEs, leading to alteration of SE-regulated gene expression (*9*). Our study also found that BET proteins at enhancers were more vulnerable to JQ1 than those at promoters despite that promoters and coding regions take up the largest fraction of BET-binding peaks. However, there was no difference in JQ1 sensitivity between BRD2/3/4-cobound SEs and TEs, and only a small number of JQ1–down-regulated genes are regulated by BET-bound SEs (Fig. 3, C and E). This indicates that BET disruption at the SE cannot be the major determinant for the JQ1 sensitivity in neurons. Genes (39.33%) whose transcription was down-regulated by JQ1 had BET binding only at promoters (Fig. 3G). Thus, the molecular mechanism of BET protein action could be cell context dependent. The local environment of a gene is also an important determinant for BET sensitivity, as reduced BET binding, especially BRD2, also occurs beyond the cis-regulatory regions of JQ1-down genes. Noted from our peak analysis was that BRD2/3/4-cobound regions were more effectively disrupted by JQ1 than the regions bound by one or two family members (Fig. 2E and fig. S4A). Although further studies will be needed to understand the exact nature of the cellular and gene context that determines the sensitivity to BET inhibition, our observation suggests that a coordinated nature of BET proteins underlies differential JQ1 sensitivity.

Recent studies in the brain reported that BET function is important for neuronal gene expression and behavior, but the beneficial effects of BET inhibition were somewhat inconclusive. For example, one study found that BET inhibition by I-BET858 in WT caused the development of autism-like syndromes, but other study showed that JQ1 improved memory performance of WT mice and also ameliorated autism phenotypes when treated in the FXS mouse model (*35*, *37*). BET inhibition in neurodegenerative disorders also showed mixed results (*36*, *38*, *39*). These mixed effects of BET inhibition could be accounted for by the experimental conditions such as the dose, duration, and the type of inhibitor used in each study but also implicate that nonspecific BET inhibition might have side effects. Our study attempted to understand the molecular nature of BET inhibition in more detail by investigating all three major BET family members in an FXS mouse model. We saw increased expression of BRD4 protein in *Fmr1* KO mice in P20 mice as reported in a previous study (*37*), but at P60, we found that the chromatin binding level of BRD4 was largely unaffected across the cis-regulatory regions except at a small number of enhancers (Fig. 7C). Unexpectedly, we saw concomitant decreases in BRD2/3 expression in *Fmr1* KO, which also caused a significant impairment in their chromatin binding. Altered bindings of individual BET proteins were largely correlated with transcriptional changes. Therefore, FXS phenotypes result from the combined consequences from the functional alterations of BRD2/3 and BRD4 in the opposite direction. According to this finding, BET inhibition alone would not be an optimal solution for treating FXS syndrome as it could exacerbate the impairment in BRD2/3 function while abnormally activated enhancers caused by additional BRD4 binding might be suppressed. Our finding of an unexpected relationship between CBP/p300 HAT activity has offered an alternative strategy for alleviating the FXS. Although HAT activity has mainly been linked to enhanced LTP (*48*) and memory formation (*49*), our study has revealed an unexpected relationship between CBP/p300 HAT activity and BET recruitment. Although CBP/p300 activity was known to be important for the bromodomain-dependent recruitment of BET proteins (*20*), we found that individual family members were differentially affected by the CBP/p300 small-molecule inhibitor, C646. C646-induced alterations in BET occupancy showed the opposite trend with the BET binding patterns observed in *Fmr1* KO mice except for BRD3 that showed no or little change (Figs. 6D and 7D). Given that BRD3 had a minimal impact on gene expression and behavior (Figs. 1J and 5D), restoring the occupancy patterns of BRD2 and BRD4 in FXS by C646 might be able to ameliorate the FXS syndromes. We found that the treatment of *Fmr1* KO mice with C646 significantly improved fear memory formation (Fig. 8, H to K). Our study also provides a molecular mechanism for the previous observation that CBP/p300 HAT inhibition could have a positive impact on neuronal plasticity and memory formation (*50*, *51*). Together, our findings of the coordinated interplay between BET family members have uncovered detailed molecular features of BET inhibition, providing previously unknown insight into the development of BET regulation–based therapeutic approach. Our study also suggests that the development of additional BET inhibitors that are specific for each family member would offer improved treatment options.

## MATERIALS AND METHODS

### Animals

All experiments carried out using animals were reviewed and approved by the Institutional Animal Care and Use Committee at University of Texas Southwestern Medical Center and Pohang University of Science and Technology. Congenic *Fmr1* KO mice were provided by K. M. Huber and bred on the C57BL/6J background.

### Mouse cortical neuron culture and stimulation

Mouse cortical neurons were dissected at embryonic day 16.5 (E16.5) and cultured in neurobasal media (NB) (Thermo Fisher Scientific, 21103) supplemented with 2% B-27 (Thermo Fisher Scientific, 17504) and 1% GlutaMAX (Thermo Fisher Scientific, 35050). For KCl depolarization, neurons at days in vitro (DIV) 7 were made quiescent by 1 μM TTX (Tocris, Minneapolis, MN; 1078) overnight and then added with 55 mM KCl for the indicated length of time. In BDNF stimulation experiment, neurons without TTX treatment were incubated with BDNF (10 ng/ml; R&D System, Minneapolis, MN; 248-BD-005) for the indicated length of time. For JQ1 experiment, TTX-treated neurons at DIV 7 were pretreated with ±JQ1 (0.5 μM; ApexBio Technology, Boston, MA; A8181/A1910) for 5 min and then followed by 55 mM KCl for the indicated length of time. For C646 experiment, quiescent neurons were pretreated with C646 (20 μM; Sigma-Aldrich, St. Louis, MO; SML0002) for 48 hours, followed by 55 mM KCl for 30 min.

### shRNA design, transfection, and lentivirus infections

shRNAs against *Brd2*, *Brd3*, *Brd4*, and *Cbp* were designed as described (*52*, *53*). Individual shRNAs were subcloned into the Hpa I/Xho I sites of the pLLX lentiviral vector. To generate lentivirus, lentiviral constructs containing the indicated shRNAs in the pLLX-shRNA-GFP, along with the helper plasmids Δ8.9 and vesicular stomatitis virus (VSV-G), were cotransfected into human embryonic kidney (HEK) 293T cells using polyethylenimine (PEI; 1 mg/ml; Polysciences, Warrington, PA; 23966). NB supplemented with B-27 and GlutaMAX was completely changed on the next day. Lentivirus transduction was carried out for additional 48 to 72 hours. Lentivirus containing media was filtered by a syringe filter (0.45 μm; Millipore, SLHV033RS) and directly used to infect neurons at DIV 3 and harvested at DIV 7. KD efficiency was measured by RT-qPCR and Western blotting. See table S6 for complete oligonucleotide list.

### Real-time quantitative polymerase chain reaction

Total RNA was prepared from cortical neurons at DIV 7 using TRIzol (Invitrogen, Carlsbad, CA; 15596) according to the manufacturer’s protocols. Subsequently, total RNAs were reverse transcribed into cDNA using high-capacity reverse transcription kit (Applied Biosystems, Warrington, UK; 4374967). Primers used are listed in table S6. PCR amplification conditions were previously described (*54*).

### Chromatin immunoprecipitation

ChIP assays were carried out as previously described with minor modifications (*54*). At DIV 7, cultured cortical neurons were treated with the indicated conditions and then fixed in cross-linking buffer [0.1 M NaCl, 1 mM EDTA, 0.5 mM EGTA, and 25 mM Hepes-KOH (pH 8.0)] containing 1% formaldehyde (Sigma-Aldrich, 252549) for 10 min at room temperature. Cross-linking was quenched by glycine (final 125 mM) for 5 min at room temperature and harvested in phosphate-buffered saline protease inhibitors on ice. Pelleted neurons were lysed in ice-cold buffer I [50 mM Hepes-KOH (pH 7.5), 140 mM NaCl, 1 mM EDTA (pH 8.0), 10% glycerol, and 0.5% IGEPAL CA630 and protease inhibitors] to isolate nuclei. Nuclei were sonicated in ice-cold buffer III [10 mM tris-HCl (pH 8.0), 300 mM NaCl, 0.1% sodium deoxycholate, 1% Triton X-100, 1 mM EDTA (pH 8.0), 0.5 mM EGTA (pH 8.0), and protease inhibitors]. The resulting nuclear extracts were centrifuged at 13,200 rpm for 15 min at 4°C to separate insoluble fraction. The supernatant was then incubated with anti-BRD2 (Bethyl, A302-583A), anti-BRD3 (Active Motif, 61489), or anti-BRD4 (Bethyl, A301-985A100 or rabbit antibody gifted by C.-M. Chiang) overnight at 4°C. The immune complexes were pelleted and washed twice with each of the following buffers: low salt [0.1% SDS, 1% Triton X-100, 2 mM EDTA, 20 mM tris-HCl (pH 8.1), and 150 mM NaCl], high salt [0.1% SDS, 1% Triton X-100, 2 mM EDTA, 20 mM tris-HCl (pH 8.1), and 500 mM NaCl], and LiCl buffer [250 mM LiCl, 1% IGEPAL CA630, 1% sodium deoxycholate, 1 mM EDTA, and 10 mM tris (pH 8.1)]. In each wash, the beads were incubated with wash buffer for 10 min at 4°C. The washed beads were then rinsed once with 1× TE [10 mM tris-HCl (pH 8.0) and 1 mM EDTA]. The immune complexes were eluted from the beads twice by elution buffer [10 mM tris-HCl (pH 8.0), 1 mM EDTA (pH 8.0), and 1% SDS] at 65°C for 10 min. The cross-linking was reversed by incubation at 65°C for 5 to 6 hours. The resulting eluate was treated with RNase A (10 μg; Qiagen, Hilden, Germany) for 1 hour at 37°C and proteinase K [4 U; New England Biolabs (NEB), Ipswich, MA; P8107S] for another 2 hours at 55°C. The DNA was purified by phenol:chloroform extraction (Thermo Fisher Scientific, 15593), followed by a PCR purification kit (Qiagen, Hilden, Germany; 28106). Primers are listed in table S6.

### ChIP sequencing

ChIP-seq library construction was performed using NEBNext ChIP-Seq Library Prep Master Mix Set (NEB, E6240) following the manufacturer’s instruction with modifications. Briefly, the end-repaired ChIP DNA fragments were size selected [100 to 300 base pairs (bp)], deoxyadenosine triphosphate (dATP) [dA] tailed, and then ligated with adaptors. The adaptor-ligated ChIP DNA fragments were digested by USER enzyme and amplified by 14 to 16 cycles of PCR. The amplified ChIP DNA library was size selected (250 to 350 bp) and proceeded to sequencing.

ChIP-seq libraries were sequenced on Illumina HiSeq 2500 or NextSeq 500 instrument with 50- or 75-bp single-end reads according to the manufacturer’s instructions (Illumina) by the UTSW McDermott Next Generation Sequencing Core. The FASTQ reads were aligned to UCSC’s mm10 genome using Bowtie2 with default parameters (*55*). Reads with mapping quality of less than 10 were removed using SAMtools (*56*). To normalize the differences in sequencing depths, the mapped reads were “down sampled” to the lowest number of the uniquely mapped reads with duplicates followed by duplicate reads removal using “Sambamba” (*57*). The bigWig files were generated using “bamCoverage” included in the “deepTools” package for visualization on University of California Santa Cruz (UCSC) genome browser. The coverage values in bigWig files were normalized to RPGC (reads per genomic content).

ChIP peaks were called using model-based analysis of ChIP-Seq (MACS) with parameters “--tsize=50 --gsize mm --nomodel True --shiftsize=65 --wig --space=10” against input chromatin samples as control data (*58*). Threshold for *P* value was set at 1 × 10^−9^. Threshold for the fold_enrichment was set at 10. Master peaks (or reproducible peaks) were identified only if the ChIP-seq peaks overlap more than 50% of the shortest peaks in −JQ1 samples across two replicates. To find overlapping peaks among different BET proteins and to call the differentially bound peaks under different conditions, we merged the master peaks from different samples, called “merged peaks”. Mergepeaks of Hypergeometric Optimization of Motif EnRichment (HOMER) (*59*) was used.

To generate ChIP coverage plots, we used either Ngs.plot R package (*60*) or HOMER. For the ngs.plot software, the parameter of Fragment (insert) length was set to 180, and Refseq database mm10 was used. For HOMER, “makeTagDirectory” program was used. We used BAM files that contained down-sampled duplicate removed reads to create tag directories, which contain tag information classified per chromosome wise. Using HOMER’s inbuilt Perl scripts annotatePeaks.pl and analyzeRepeats.pl, data were used to create coverage plots.

### Identification of SEs and TEs

SEs and TEs were identified according to MACS-called H3K27ac-enriched peaks. First, H3K27ac peaks were identified in p56 tissue (GSM1264366-GSM1264369) (*61*) and KCl-stimulated neurons (GSM1467414-GSM1467419) (*62*) using MACS (*58*). Mapped reads of two biological replicates in each condition group were merged before the SE calling analysis using Bamtools (*63*). Then, the ranking of super-enhancer (ROSE) algorithm (*9*) was used to define SEs with the identified H3K27ac peaks. A line with a slope of one tangent to the curve is used as a cutoff to distinguish SEs above the point and TEs below the point of tangency. SEs are defined as the population of enhancers above the inflection point of the curve. The H3K27ac peaks that were not overlapped with the SE or promoter regions of known genes were defined as TEs. SE- or TE-regulated genes were defined as genes within 50 kb upstream and downstream of SEs or TEs. SE- or TE-based scaled plots were generated using deepTools (*64*).

### mRNA sequencing

mRNA-seq library was constructed using the TruSeq RNA Library Preparation Kit (Illumina) according to the manufacturer’s instructions. FASTQ reads from UTSW Sequencing Core were mapped to UCSC’s mm10 genome using TopHat (*65*) with options “-a 8 -m 0 -I 500000 -p 8 -g 20 --library-type fr-firststrand --no-novel-indels --segment-mismatches 2”. Since these data were strand specific, we used “-library-type fr-firststrand” option from TopHat. Reads with low mapping quality (<10) were removed using SAMtools (*56*). Duplicate reads were marked by Picard MarkDuplicates (https://broadinstitute.github.io/picard/). Tag directories for each sample were created using the “makeTagDirectory” program. RNA expression was quantified using HOMER’s inbuilt Perl script “analyzeRepeats.pl.” These scripts offer flexibility to calculate expression values as reads per kilobase per million mapped reads (RPKM) normalized to 10 million at introns, exons, and gene body locations. “makeUCSCfile” from HOMER was used to create bedGraph files at 1-bp resolution and created bigWig files for visualization on UCSC genome browser. All coverage values were normalized to 10 million reads.

We set expressed gene criteria as “RPKM values higher than 1 at least in 1 of 12 JQ1-related samples (six conditions, two replicates for each condition).” We identified 12,723 “expressed” genes. The subsequent RNA-seq analyses were performed with these 12,723 genes. To call significant DEGs, we set our criteria as “FC of RPKM is more than 1.5 and FDR of DESeq2 is less than 0.05 in both replicates.” To identify +JQ1 or KCl-dependent genes, we took genes that were significantly changed at either time point (1 or 3 hours).

### Global run-on sequencing

Ten million nuclei per sample were used for global run-on, and base hydrolysis was performed as previously described (*66*). Nascent RNA was immunoprecipitated with anti-BrdU antibody-conjugated beads (Santa Cruz Biotech, Santa Cruz, CA; sc-32323AC). Purified run-on RNA was subjected to polyA tailing by poly(A)polymerase (14.06 U; NEB, M0276) for 12 min at 37°C. PolyA-tailed RNA was subjected to another round of immunopurification by using anti-BrdU antibody-conjugated beads. Reverse transcription was then performed using SuperScript III Reverse Transcriptase (200 U; Thermo Fisher Scientific, 18080) with RT primer (pGATCGTCGGACTGTAGAACTCT/idSp/CCTTGGCACCCGAGAATTCCATTTTTTTTTTTTTTTTTTTTVN) for 2 hours at 48°C. Extra RT primers were removed by Exonuclease I (100 U; NEB, M0293) for 2 hours at 37°C. cDNAs were fragmented with basic hydrolysis and size selected (130 to 500 nucleotides) in a 6 to 8% polyacrylamide tris-borate-EDTA (TBE)–urea gel. Purified cDNAs were circularized using CircLigase (50 U; Epicentre, CL4111K) for 2 hours at 60°C and relinearized at the basic dSpacer furan with Ape 1 (15 U; NEB, M0282) for 2 hours at 37°C. The relinearized single-stranded DNA template was subjected to PCR amplification by using barcoded primers for Illumina TrueSeq small RNA sample and Phusion High-Fidelity DNA Polymerase (2 U; NEB, M0530). Subsequently, PCR products were size-selected in 6% polyacrylamide TBE gel (175 to 400 bp) and purified. The final libraries were sequenced using Illumina NextSeq 500 per the manufacturer’s instructions.

For analysis, the raw FASTQ reads were trimmed using cutadapt with parameters -a AAAAAAAAAAAAAAAAAAAA -z -e 0.10 -f fastq -m 32 (*67*). The reads were then submitted to Burrows-Wheeler aligner (BWA) for mapping to the mm10 UCSC annotation. SAMtools and the HOMER package were used to make visualization tracks and RPKM calculations. RPKM was calculated by normalizing to 10 million reads. We set expressed gene criteria as “RPKM is more than 0.5 at least in 1 of 20 samples (10 conditions, two replicates for each condition).” We identified 12,974 expressed genes. The subsequent GRO-seq analyses were done with these 12,974 genes. To call significant DEGs, we set our criteria as “fold change of RPKM is more than 1.5 and FDR of DESeq2 (*68*) is less than 0.05 in both replicates.” See table S3 for DEGs.

### Rapid immunoprecipitation mass spectrometry of endogenous proteins

RIME was performed as previously described (*41*) except that cortical neurons at DIV 7 were subject to the ChIP procedure described above. Precipitated protein complexes were boiled for 10 min in SDS sample buffer and separated by SDS–polyacrylamide gel electrophoresis (PAGE). Gel bands were digested overnight with trypsin (Pierce), followed by destaining, reduction with dithiothreitol, and alkylation with iodoacetamide (Sigma-Aldrich). The samples then underwent solid-phase extraction cleanup with an Oasis HLB plate (Waters), and the resulting samples were injected onto an Orbitrap Fusion Lumos mass spectrometer coupled to an Ultimate 3000 RSLCnano liquid chromatography system. Samples were injected onto a 75-μm i.d., 75-cm long EasySpray column (Thermo Fisher Scientific) and eluted with a gradient from 0 to 28% buffer B over 90 min. Buffer A contained 2% (v/v) acetonitrile (ACN) and 0.1% formic acid in water, and buffer B contained 80% (v/v) ACN, 10% (v/v) trifluoroethanol, and 0.1% formic acid in water. The mass spectrometer operated in positive ion mode with a source voltage of 1.8 kV and an ion transfer tube temperature of 275°C. MS scans were acquired at 120,000 resolution in the Orbitrap, and up to 10 tandem MS (MS/MS) spectra were obtained in the ion trap for each full spectrum acquired using higher-energy collisional dissociation for ions with charges 2 to 7. Dynamic exclusion was set for 20 s after an ion was selected for fragmentation.

Raw MS data files were analyzed using Proteome Discoverer v2.2 (Thermo Fisher Scientific), with peptide identification using Sequest HT searching against the mouse protein database from UniProt. Fragment and precursor tolerances of 10 parts per million and 0.6 Da were specified, and three missed cleavages were allowed. Carbamidomethylation of Cys was set as a fixed modification, with oxidation of Met set as a variable modification. Five percent of FDR cutoff was used to determine enriched polypeptides. Immunoglobulin G IP control was used to exclude nonspecifically associated polypeptides. PSMs indicate the number of peptide spectrum matches or the number of spectra assigned to peptides that contributed to the inference of the protein. Abundance indicates the sum of the peak intensities for each peptide identified for that protein.

### Western blot

Protein extracts from cortical neurons or tissues were prepared with sample buffer [60 mM tris-HCl (pH 6.8], 2% sodium dodecyl sulfate, 10% glycerol, 5% 2-mercaptoethanol, and 0.1% bromophenol blue] and boiled for 5 min. For FACS sorting, the whole cortex dissected from WT or *Fmr1* KO was homogenized by douncing followed by nuclei extraction via sucrose gradient ultracentrifugation (*69*). The nuclei were recovered from the pellet, resuspended, and incubated with NeuN antibody (Millipore, MAB377). Immunotagging with anti-NeuN conjugated to Alexa Fluor 488 (Invitrogen, A-21202) allows for sorting of the NeuN^+^ neuronal nuclei by fluorescence-activated sorting through a FACS machine (MoFlo Astrios, Beckman Coulter), followed by Western blotting. Proteins were separated by SDS-PAGE and subjected to Western analysis using the following antibodies: anti-BRD2 (Bethyl, A302-583A), anti-BRD3 (Active Motif, 61489) or anti-BRD4 (Bethyl, A301-985A100), anti-CBP (Santa Cruz, sc-369x), anti-FMRP (Cell Signaling, 4317S), anti–β-actin (Santa Cruz, sc-47778), anti–α-tubulin (Cell Signaling, 2144S), anti-H3K27ac (Abcam, Ab4729), anti-H4K8ac (Abcam, Ab15823), anti-H3K4me1 (Abcam, Ab8895), anti-H3ac (Millipore, 06-599), or anti-H3 (Abcam, ab176842).

### Immunoprecipitation analysis

IP analysis was performed as previously published (*52*) with minor modifications. Cortical neurons at DIV 6 to 7 were made quiescent overnight in 1 μM TTX and then KCl (55 mM) depolarized for 30 min. Neurons were lysed in ice-cold lysis buffer [50 mM Hepes-KOH (pH 7.5), 140 mM NaCl, 1 mM EDTA (pH 8.0), 10% glycerol, and 0.5% IGEPAL CA630 and protease inhibitors]. Nuclei were pelleted by centrifugation at 3000 rpm for 5 min at 4°C and then added with ice-cold extraction buffer [10 mM tris-HCl (pH 8.0), 600 mM NaCl, 1.5 mM MgCl_2_, 0.05% sodium deoxycholate, 0.5% Triton X-100, 1 mM EDTA (pH 8.0), 0.5 mM EGTA (pH 8.0), and protease inhibitors]. Nuclear extract was sonicated briefly and incubated for 10 min on ice. The resulting extract was diluted to a final concentration of 100 mM NaCl and cleared by centrifugation at 13,200 rpm for 15 min at 4°C. The supernatant was incubated with the indicated antibodies at 4°C for overnight with rocking and followed by Protein A/G PLUS Agarose (Santa Cruz, sc-2003) incubation for 2 hours at 4°C. The immune complexes were precipitated and washed three times with extraction buffer and boiled for 5 min in SDS sample buffer. The samples were separated by SDS-PAGE and subjected to immunoblot analysis.

### AAV and stereotaxic injection

The shRNAs were cloned into the Bam HI/Eco RI sites of the pAAV-U6-shRLuc-CMV-ZsGreen vector (Penn Vector Core, P0111). To generate AAV, pAAV-U6-CMV-ZsGreen, along with the Helper and AAV-DJ plasmids, were transfected into HEK293T cells using PEI (1 mg/ml) for 3 days. Virus purification was performed as previously described with minor modifications (*70*). Briefly, cells were harvested and incubated in the presence of 0.5% sodium deoxycholate and benzonase (50 U/ml) (Sigma-Aldrich, E1014) at 37°C for 1 hour. The virus was purified using an iodixanol step gradient and concentrated using an Amicon Ultra centrifugal filter device [100 K nominal molecular weight limit (NMWL), Millipore, UFC910008]. The genomic titer of each virus was determined by qPCR. Male C57BL/6J mice at 8 weeks of age were anesthetized with tribromoethanol (200 mg/kg; Sigma-Aldrich, T48402) (*53*). AAV (0.6 μl per hemisphere) was bilaterally injected into the dorsal hippocampal CA1 region using a glass pipette with the following coordinates: anteroposterior, +1.90 mm; medial lateral, ±1.25 mm; dorsal ventral, −1.20 mm. Animals were subjected to fear conditioning 2 weeks after stereotaxic injection.

### Behavior experiments

Male mice at 8 to 10 weeks of age were housed in light 12 hours: dark 12 hours (LD12:12) conditions. Mice were adapted to handling and transportation procedures once a day for 3 to 4 days before the experiment. On the day of training, mice were transported to the behavior room at least 1 hour before the experiment. Vehicle or +JQ1 (12.5 mg/kg; ApexBio Technology) was administrated intraperitoneally 15 min before fear conditioning. Vehicle or C646 (15 mg/kg; Sigma-Aldrich, SML0002) was administrated (intraperitoneally) 2 days before fear conditioning. AAV-injected mice were subjected to fear conditioning after 2 weeks. Mice were briefly anesthetized by isoflurane for intraperitoneal injection to minimize the stress induced by injection.

For fear conditioning, mice were placed in the fear conditioning chamber and allowed to explore for 3 min (acclimation). Mice were then received a 2-s shock (0.86 mA) followed by a 1-min pause. This was repeated a total of four times, followed by the final 1-min pause. Mice were then returned to their home cage. For some experiments, mice were euthanized 1 hour after fear conditioning, and brain tissues were taken out for downstream experiments. For the behavioral (freezing) test, 24 hours after fear conditioning, the mice were placed back into the same chamber, and freezing behavior was monitored via video camera and recorded every 10 s for 3 min with Freezeframe 4 software.

### Statistics

Statistical analysis was performed using two-tailed unpaired *t* test with Welch’s correction or Mann-Whitney *U* test for comparison between two groups of parametric or nonparametric samples. For some datasets, two-tailed paired *t* test was used for comparison between two groups of parametric samples. Kruskal-Wallis test was also used for comparison between unmatched nonparametric data. Statistical significance between cumulative probability graphs was determined by the Kolmogorov-Smirnov test. Hypergeometric tests were performed in DynaVenn (https://ccb-compute.cs.uni-saarland.de/dynavenn) implemented in Python for analysis of overlaps of gene groups. Bar plots show mean values and error bars for bar plots are SEM. No statistical methods were used to predetermine sample size, but our sample sizes are similar to those generally used in the field. Randomization and blinding were not used. Mice were used according to their genotype. We considered *P* < 0.05 to be statistically significant (*P < 0.05, **P < 0.01, and ***P < 0.001).

## SUPPLEMENTARY MATERIALS

Supplementary material for this article is available at http://advances.sciencemag.org/cgi/content/full/7/21/eabf7346/DC1

## Appendix

View/request a protocol for this paper from *Bio-protocol*.
